# Data of ascending cortical vein occlusion induced spreading depression

**DOI:** 10.1016/j.dib.2018.04.042

**Published:** 2018-04-18

**Authors:** Buket Dönmez-Demir, Muge Yemisci, Turgay Dalkara

**Affiliations:** a Institute of Neurological Sciences and Psychiatry, Hacettepe University, Ankara, Turkey; b Department of Neurology, Faculty of Medicine, Hacettepe University, Ankara, Turkey

**Keywords:** Cortical spreading depression, Migraine, Cortical veins, Venular occlusion

## Abstract

The data presented in this article are related to the research article entitled “Microembolism of single cortical arterioles can induce spreading depression and ischemic injury; a potential trigger for migraine and related MRI lesions” (Donmez-Demir et al., 2018) [1]. This article presents data showing that thrombosis of a small ascending cortical vein (25 µm) in the mouse may also trigger spreading depression as does penetrating arteriole occlusion, although less frequently (22% vs. 100%).

**Specifications Table**

**Table:** 

Subject area	*Neuroscience*
More specific subject area	*Migraine*
Type of data	*DC potential recording, Laser speckle contrast imaging of cortical blood flow*
How data was acquired	*Stereo and upright microscope, Laser speckle contrast imaging (LSCI), Electrophysiological recording (ADI Instruments)*
Data format	*Raw and analyzed*
Experimental factors	*Mice were anesthetized, the skull was thinned and a burr hole was opened over the parietal bone*
Experimental features	*An ascending vein surfacing the cortex was thrombosed with FeCl*_*3*_*and CSD generation was monitored by electrophysiological recording and LSCI.*
Data source location	*Hacettepe University, Ankara, Turkey*
Data accessibility	*The data are available in this article*
Related Research Article	*Microembolism of single cortical arterioles can induce spreading depression and ischemic injury; a potential trigger for migraine and related MRI lesions.*
	*Dönmez-Demir et al.*[1]

**Value of data**

● Venous hypoperfusion had not previously been considered in the pathophysiology of migraine until very recently [2] although headache is seen in patients suffering from thrombosis of large veins or venous malformations.
● Here, we show that CSD, the putative cause of migraine aura, is generated upon thrombosis of a small cortical ascending vein (about 25 µm in radius) but less frequently compared to arteriolar occlusion.
● This finding conforms to the reports showing that, in the mouse cortex, ascending veins can oxygenate the tissue encircling them although to a lesser extent than do arterioles [3], [4].

## 1 Data

We found that thrombotic occlusion of a single ascending cortical vein with FeCl_3_ at the point they surfaced over the cortex (before joining surface veins) induced a CSD, however, much less frequently (in 2 out of 9 mice) compared to penetrating arteriole occlusions, which always trigger a CSD [1]. These CSDs shared the same features with CSDs induced by arteriolar occlusion as well as with CSDs evoked by pinprick at the end of each experiment. Additionally, we observed multiple (2–6 in half an hour) small-amplitude (mini) CSDs (less than 1 mV) in 6 out of 9 mice. To make sure that low amplitudes were not caused by poor electrical contact of the extracranial electrodes with the skull, we also recorded intracortically in a mouse subjected to ascending vein thrombosis and observed 6 mini CSDs of 2–3.3 mV without any full-blown CSDs. We used urethane anesthesia during recordings to minimize the anesthetic-induced inhibition of CSD.

## 2 Experimental design, materials and methods

### 2.1 Experimental animals and protocol

Animal housing, care, and experimental manipulations were approved by Hacettepe University Ethics Committee (2011/18 and 2013/66) and performed in accordance with institutional replace, reduce and refine guidelines. Male Swiss albino mice (weighing 25–35 g) were obtained from Hacettepe University Experimental Animal Facility, housed in standard cages under diurnal lighting conditions (12 h darkness and 12 h light). Mice (*n* = 10) were placed in a stereotaxic frame under isoflurane anesthesia, which was replaced by urethane (1.25 g/kg, i.p.) and xylazine (10 mg/kg, i.p.) after surgery and the animals were supplemented with oxygen 2 l/min throughout the surgery and experiment. Body temperature was maintained at 37.0 ± 0.2 °C by a feedback-regulated homoeothermic blanket and rectal probe. Heart rate and tissue oxygen saturation were continuously monitored using a pulse oximeter with a mini Y clip attached to one of the lower extremities. Tissue O_2_ saturation was kept over 95%.

**Fig. 1:**
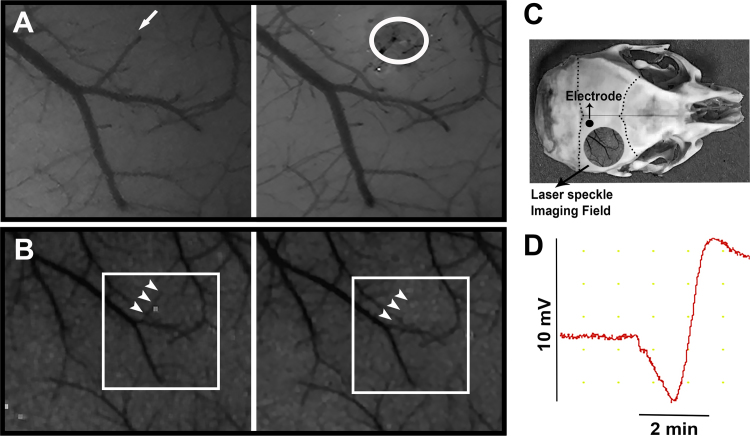
Thrombosis of an ascending vein induces CSD. **A.** The upper panels show the pial arterioles and venules imaged through the thinned skull with a stereomicroscope. Venules can be differentiated from arterioles by their darker color and less bright vessel wall reflection. The arrow marks a venule surfacing over the cortical surface and then joining a larger vein. White circle denotes the same venule thrombosed by FeCl_3_ topically applied with a micropipette at the point where the vein surfaced. **B.** Laser speckle contrast images of the same area in the lower row show that the blood flow within the same venule disappeared after thrombotic occlusion (arrows). These images have lower magnification because LSCI images were acquired with low resolution. **C.** Illustrates the sites where blood flow changes were recorded with laser speckle contrast imaging (LSC) and electrophysiological recording was made with electrode through the intact thinned mouse skull. **D.** The trace on the right shows the DC potential recording over the intact skull and illustrates a spreading depression wave triggered after occlusion of the venule.

Head skin was opened after a midline incision under a stereomicroscope. A 3 × 3 mm part of skull over the right hemisphere between the bregma and lambda was thinned to visualize the vessels and then a small burr hole was opened with help of laminectomy forceps to apply the FeCl_3_ filled micropipette (Fig. 1). The skull was irrigated with cool artificial cerebrospinal fluid (aCSF) to reduce heating caused by drilling procedure. Dura under the burr hole was kept intact and maintained moist by repeated applications of aCSF preheated to 37 °C. CSD generation and the associated cortical blood flow changes were monitored by LSCI and electrophysiological recording (Fig. 1).

### 2.2 Ascending vein occlusion

A glass micropipette having a tip diameter of 40–50 μm was filled with 30% FeCl_3_ by using a microfil. An ascending cortical vein that is away from other vessels was targeted where it surfaced the brain (Fig. 1). To avoid spilling of FeCl_3_, the dura was air-dried. The micropipette tip was gently touched the dura over the targeted vein for 10 min with help of a micromanipulator to induce thrombosis. The micropipette was pulled back and the dura was instantly washed with artificial CSF at 37 °C.

### 2.3 Electrophysiological recordings

The direct current (DC) potentials were recorded by Ag-AgCl pellet electrodes (Warner Instruments, E205, 1 mm in diameter). Recordings were obtained over the thinned skull posterior to the burr hole (Fig. 1). EEG gel was applied to the electrode tip to maintain good electrical contact. The ground lead (a Ag-AgCl-plated disk electrode) was placed between the neck muscles. Gold pins and jacks (Warner Instruments, WC1–10 and PJ1–10) were used to connect the electrodes to a data acquisition system (Powerlab 16/35, ADInstruments). Digitized signals were stored on a computer.

### 2.4 Laser speckle contrast imaging

Laser Speckle Contrast images were captured using a CCD camera (Basler 602F, Basler Vision Technologies, Ahrensburg, Germany) attached to a stereomicroscope with 1×, long working distance lens (Nikon SMZ 1000) and by custom-developed software (courtesy of Dr. A.K. Dunn, University of Texas at Austin). The cortical surface through the thinned skull was diffusely illuminated at 785 nm wavelength by a laser diode (Thorlabs). LSCI images were captured every 10 s during 1-h experiment and processed as described previously [5].
