# Supplementary material for: Data of ascending cortical vein occlusion induced spreading depression
Source: Data Brief. 2018 Apr 18;18:1462–5. doi: 10.1016/j.dib.2018.04.042 (PMC5997957; doi:10.1016/j.dib.2018.04.042)
Supplement: Supplementary file 1 — Transparency document [file mmc1.docx]

**Conflict of interest**

All the author confirms no conflict of Interest.
